# The paradox of Zeno in bariatric surgery weight loss: Superobese patients run faster than morbidly obese patients, but can't overtake them

**DOI:** 10.3389/fsurg.2023.1100483

**Published:** 2023-02-02

**Authors:** Fabio Medas, Enrico Moroni, Simona Deidda, Luigi Zorcolo, Angelo Restivo, Gian Luigi Canu, Federico Cappellacci, Pietro Giorgio Calò, Stefano Pintus, Giovanni Fantola

**Affiliations:** ^1^ Department of Surgical Sciences, University of Cagliari, Cagliari, Italy; ^2^ Obesity Surgery Unit, Surgical Department, “ARNAS G. Brotzu” Hospital, Cagliari, Italy

**Keywords:** obesity surgery, superobesity, morbidly obese patients, laparoscopic sleeve gastrectomy, laparoscopic Roux-and-Y gastric bypass

## Abstract

**Introduction:**

Superobesity (SO) is defined as a BMI > 50 Kg/m^2^, and represents the extreme severity of the disease, resulting in a challenge for the surgeons.

**Methods:**

In this retrospective study we aimed to compare the outcomes of SO patients compared to morbidly obese (MO) patients.

**Results:**

We included in this study 154 MO patients, with a median preoperative BMI of 40.8 kg/m^2^, and 19 SO patients with median preoperative BMI of 54.9 kg/m^2^. The MO patients underwent sleeve gastrectomy (SG) in 62 (40.3%) cases, laparoscopic Roux-and-Y gastric bypass (LRYGBP) in 85 (55.2%) cases and One-Anastomosis Gastric Bypass (OAGB) in 7 (4.5%) cases. underwent OAGB. The patients in the SO group were submitted to SG in 11 (57.9%) cases, LRYGBP in 5 (26.3%) cases, and OAGB in 3 (15.8%). At 24-month follow-up, an excess weight loss (EWL) >50% was achieved in 129 (83.8%) patients in the MO group and in 15 (78.9%) in the SO group (*p* = 0.53). A BMI < 35 kg/m^2^ was achieved in 137 (89%) patients in the MO group and from 8 (42.2%) patients in the SO group (*p* < 0.001). The total weight loss was significantly directly related to the initial BMI. Superobesity was identified as independent risk factor for surgical failure when considering the outcome of BMI < 35 kg/m^2^.

**Discussion:**

Our study confirms that, although SO patients tend to gain a greater weight loss than MO patients, they less frequently achieve the desired BMI target. In this setting, it should be necessary to re-consider malabsorptive procedures as first choice.

## Introduction

Super obesity (SO) is defined as a Body Mass Index (BMI) > 50 kg/m^2^, and represents the extreme severity of the disease, resulting in an increase in morbidity and mortality, and in poorer quality of life compared with morbid obesity (BMI > kg/m^2^ 35 and <50 kg/m^2^).

Although bariatric surgery is proven to exceed medical treatment regarding weight loss and obesity-related morbidity resolution (1), in SO patients bariatric surgery may present suboptimal and less predictable results (2, 3). Furthermore, in this class of patients, surgery presents an increase in operative risks and is technically challenging (2, 4), Conventional methods, such as Laparoscopic Roux-and-Y Gastric Bypass (LRYGBP) and Laparoscopic Sleeve Gastrectomy (SG), gain different results in SO patients, with different weight-loss trends during the first two years after surgery (2), Conversely, One-Anastomosis Gastric Bypass (OAGB) seems to result in better middle- and long-term weight-loss (4, 5), Another important issue is to define success, insufficient weight loss or weight regain after surgery (6–9); indeed SO patients frequently need revision or second step surgery to achieve optimal results.

Several studies have demonstrated that after bariatric surgery less weight was achieved at 12–24 months (10–12); thus, 24 months should be considered the threshold to define a surgical result.

Excess Weight Loss (EWL)% represents a common measure used to define the success of bariatric interventions: surgical success is defined by an EWL greater than 50% (EWL > 50%) (7). Usually, EWL > 50% is achieved more easily in morbidly obese (MO) than SO patients, independent of the surgical procedure. Another target commonly used to define surgical success, particularly in SO patients, is represented by a BMI <35 kg/m^2^.

In this study, we aimed to evaluate the outcomes of bariatric surgery in SO patients compared to MO patients, and to assess patients' and surgical factors associated with bariatric success.

## Patients and methods

### Study design

This is a unicentric, retrospective study. Data were extracted from a prospectively maintained database of all morbidly obese patients undergoing bariatric surgery in our institution.

### Inclusion criteria

We included in our study all the patients who underwent bariatric surgery from January 2018 to July 2020 at our institution. The indications for bariatric surgery were assigned according to IFSO criteria (13, 14), and all the cases were discussed and endorsed in a local interdisciplinary consensus meeting comprising surgeons, endocrinologists, nutritionists, and psychologists.

### Exclusion criteria

All patients who underwent revisional surgery and patients without at least 12 and 24 months of postoperative follow-up were excluded.

### Preoperative evaluation

Preoperative clinical evaluation was performed to detect patient characteristics (sex, age), biometric values (weight, height and BMI), and comorbidities, including diabetes, metabolic, cardiac or cerebrovascular diseases, gastroesophageal reflux disease (GERD), arthrosis and Obstructive Sleep Apnea Syndrome (OSAS). Serological blood tests according to IFSO guidelines (14), lower US-doppler and esophagogastroscopy with research of Helicobacter pylori infection were performed in all patients. Pneumological counselling, spirometry and polysomnography were performed in cases of positive pneumological disease or in cases of a score at stop-bang test ≥3. A nutritional evaluation was performed in all the patients to obtain preoperative weight loss, according to the ERAbS (Enanched Recovery after bariatric Surgery) protocol (15) and to reduce anaesthesia and surgery-associated risks. Psychological evaluation was performed to identify a history of eating disorder/behavior, anxiety or depression.

### Type of procedure

LRYGBP was usually recommended in patients who suffered from severe GERD or type 2 diabetes (T2DM), or in patients with severe sweet eating behavior. SG was the favourite procedure in case of patients younger than 30 years old, large incisional hernia or severe small bowel adhesions. OAGB was not performed routinely and was indicated only in patients with severe dyslipidemia or diabetes.

LRYGBP was performed with the creation of a gastric pouch of approximately 30 cc, with a biliopancreatic limb of 75 cm, and an alimentary limb of 100–150 cm. SG was performed with gastric section 5 cm from the pylorus and 40 Fr bougie. OAGB was performed and 40 Fr bougie gastric pouch (>10 cm) and 150–180 cm biliopancreatic limb.

### Follow-up

All patients were evaluated 1,3, 6, 12, 18, 24 months after surgery. Postoperative assessment included clinical, nutritional, or psychological evaluation, and serological blood test.

### Endpoints

The primary endpoint was surgical success at 24 months, defined as a) BMI < 35 kg/m^2^, and b) EWL > 50%.

As secondary endpoints were evaluated as predictive and protective factors for surgical success (EWL < 50% and BMI < 35 kg/m^2^).

### Statistical analysis

Patients were divided into two groups according to preoperative BMI (>50 kg/m^2^ and ≤50 kg/m^2^). The Chi-squared test or the Fisher's exact test was used to compare categorical variables, while Student's *t*-test or Mann–Whitney's *U* test was employed for continuous variables based on the data distribution. Data are presented as medians with 95% confidence intervals or means with standard deviations (SD) as appropriate.

An exploratory analysis was conducted to identify any correlations and redundancies between independent variables and to choose what models to test should be made. Multivariate logistic regression was used to evaluate factors associated with surgical failure, defined as EWL > 50% and BMI < 35 kg/m^2^ at 24 months. The correlation coefficient was calculated to determine whether the TWL was significantly associated with superobese or morbid obese status.

Statistical significance was defined as *p* < 0.05. Statistical analyses were performed with MedCalc version 20.105.

## Results

A total of 332 patients underwent primary bariatric surgery during the study period. Among them, 173 (52.1%) patients had a complete postoperative follow-up at 12 and 24 months after surgery and were then included in the study.

The MO group included 154 patients with a mean age of 45.4 ± 11.2 years and median preoperative BMI of 40.8 kg/m^2^. The SO group included 19 patients with a mean age of 41.6 ± 10.5 years and median preoperative BMI of 54.9 kg/m^2^.There were 30 (19.5%) male patients in the MO group and 4 (21.1%) in the SO group. Regarding comorbidities, OSAS was present in 52 (33.8%) patients in the MO group and in 11 (57.9%) the SO group, high blood pressure in 82 (53.2%) cases in the MO group and in 8 (42.1%) in the SO group, osteoarthrosis in 93 (60.4%) patients in the MO group and in 13 (68.4%) in the SO group, GERD in 70 (45.5%) in the MO group and in 4 (21.1%) in the SO group, diabetes mellitus in 23 (14.9%) in the MO group and in 3 (15.8) in the SO group. In univariate analysis, preoperative weight and BMI were significantly higher in the SO groups (*p* < 0.001), and the incidence of OSAS and osteoarthrosis was higher in SO group (*p* = 0.0398 and *p* = 0.0404, respectively). Full preoperative data, including comorbidities, are reported in Table 1.

**Table 1:** Preoperative features.

	MO patients (*n* = 154)	SO patients (*n* = 19)	*p*
Sex male	30 (19.5%)	4 (21.1%)	1
Age, years	45.4 ± 11.2	41.6 ± 10.5	0.1495
Preoperative BMI, Kg/m^2^ (median, IQR)	40.8 (38.1–44.4)	54.9 (53.5–58.4)	<0.001
Preoperative weight, Kg	107.1 ± 15.3	145.5 ± 18.7	<0.001
Binge eating disorder	44 (28.6%)	6 (31.6%)	0.5642
Nibbling disorder	68 (44.2%)	11 (57.9%)	0.2197
Major depressive syndrome	35 (22.7%)	6 (31.6%)	0.3933
OSAS	52 (33.8%)	11 (57.9%)	0.0398
Hypertension	82 (53.2%)	8 (42.1%)	0.3605
Arthrosis	93 (60.4%)	13 (68.4%)	0.0404
Hiatal hernia	42 (27.3%)	4 (21.1%)	0.7839
GERD	70 (45.5%)	4 (21.1%)	0.0504
Diabetes mellitus	23 (14.9%)	2 (10.5%)	1
Dyslipidemia	60 (39%)	3 (15.8%)	0.0744

MO, morbidly obese; SO, superobese; BMI, body mass index; OSAS, obstructive sleep apnea syndrome; GERD, gastroesophageal reflux disease.

As reported in Table 2, among the MO group, 62 (40.3%) patients underwent SG, 85 (55.2%) underwent LRYGBP and 7 (4.5%) underwent OAGB. The patients in the SO group were submitted to SG in 11 (57.9%) cases, LRYGBP in 5 (26.3%) cases, and OAGB in 3 (15.8%). The mean postoperative stay was 3.4 ± 1.5 days in the MO group and 3 ± 1.2 in the SO group. Postoperative complications were observed in 21 (13.6%) patients in the MO group and in 2 (10.5%) cases the in SO group.

**Table 2:** Surgical management and postoperative complications.

	MO patients (*n* = 154)	SO patients (*n* = 19)	*p*
Surgical procedure			0.0217
SG	62 (40.3%)	11 (57.9%)	
RYGBP	85 (55.2%)	5 (26.3%)	
OAGBP	7 (4.5%)	3 (15.8%)	
Postoperative stay	3.4 ± 1.5	3 ± 1.2	0.2229
Complications	21 (13.6%)	2 (10.5%)	1

MO, morbidly obese; SO, superobese; SG, sleeve gastrectomy; RYGBP, Laparoscopic Roux-and-Y Gastric Bypass; OAGBP, one-anastomosis gastric bypass.

**Figure 1:**
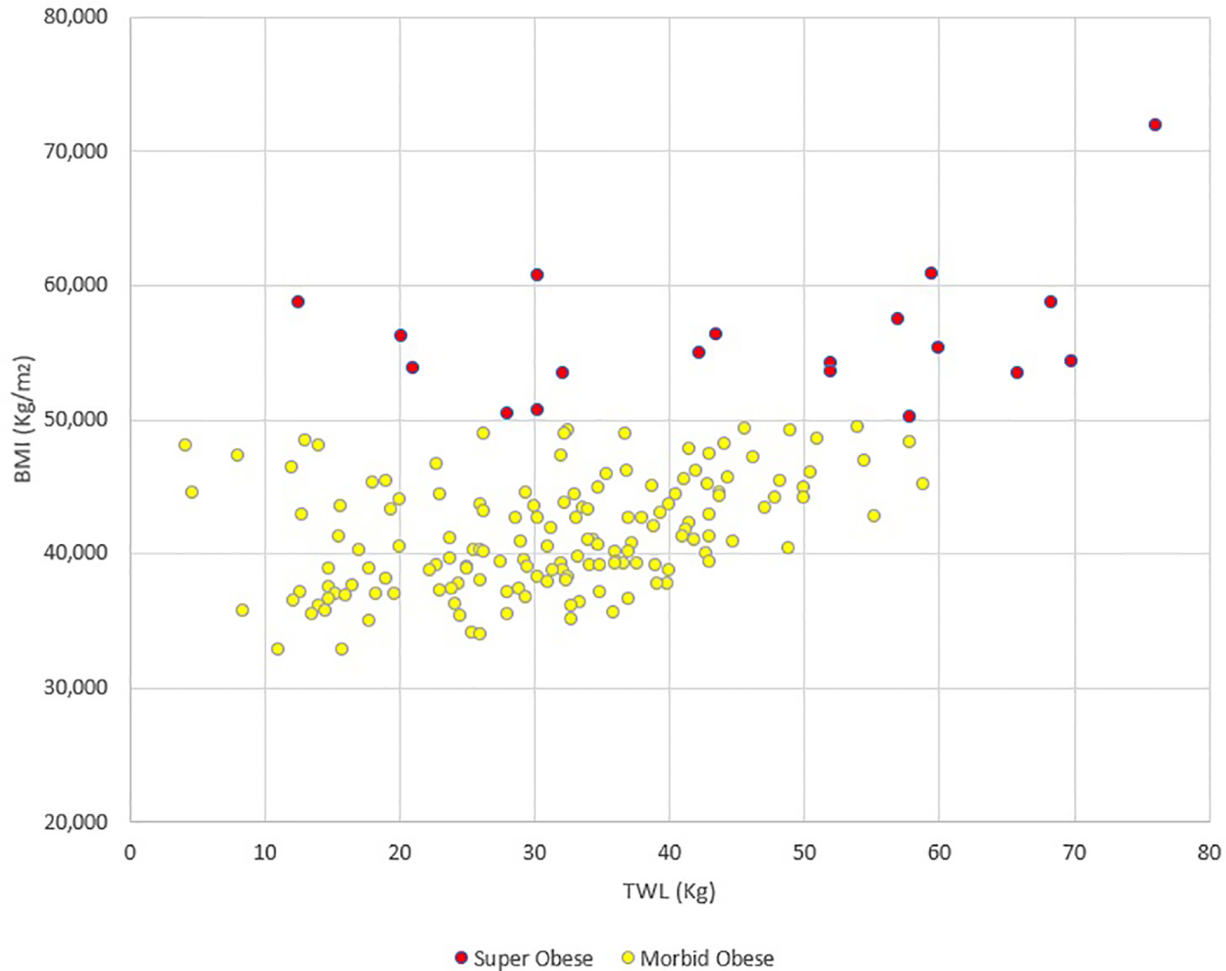
Scatter diagram representing the correlation between preoperative BMI and total weight loss (TWL). The correlation test demonstrated that preoperative BMI > 50 Kg/m^2^ was significantly associated with a higher TWL (correlation coefficient = 0.4914; *p* < 0.001).

The outcomes after 24 months of follow-up are reported in Table 3. The median BMI was 28.7 kg/m^2^ in the MO group and 36.4 kg/m^2^ in the SO group (*p* < 0.001). The TWL was 31.1 ± 11.7 Kg in the MO group, and 51.9 ± 18.3 Kg in the SO group (*p* < 0.001). The correlation test demonstrated that SO was significantly associated with a higher TWL (correlation coefficient = 0.4914; *p* < 0.001); the scatter diagram is reported in Figure 1.

**Table 3:** Results at 24-month follow-up.

	MO patients (*n* = 154)	SO patients (*n* = 19)	*p*
TWL, Kg	31.1 ± 11.7	51.9 ± 18.3	<0.001
EWL > 50%	129 (83.8%)	15 (78.9%)	0.5301
BMI < 35 Kg/m^2^	137 (89%)	8 (42.1%)	<0.001
BMI < 30 Kg/m^2^	90 (58.4%)	1 (5.3%)	<0.001
BMI, Kg/m^2^ (median, IQR)	28.7 (26–31.7)	36.4 (31.9–40.1)	<0.001

MO, morbidly obese; SO, superobese; TWL, total weight loss; EWL, Excess weight loss; BMI, body mass index.

When considering an EWL > 50%, surgical success was achieved in 129 (83.8%) patients in the MO group and in 15 (78.9%) in the SO group (*p* = 0.53). A BMI < 35 kg/m^2^ was achieved in 137 (89%) patients in the MO group and from 8 (42.2%) patients in the SO group (*p* < 0.001); a BMI < 30 kg/m^2^ was reached from 90 (58.4%) patients in the MO group and in 1 (5.3%) case in the SO group (*p* < 0.001).

**Figure 2:**
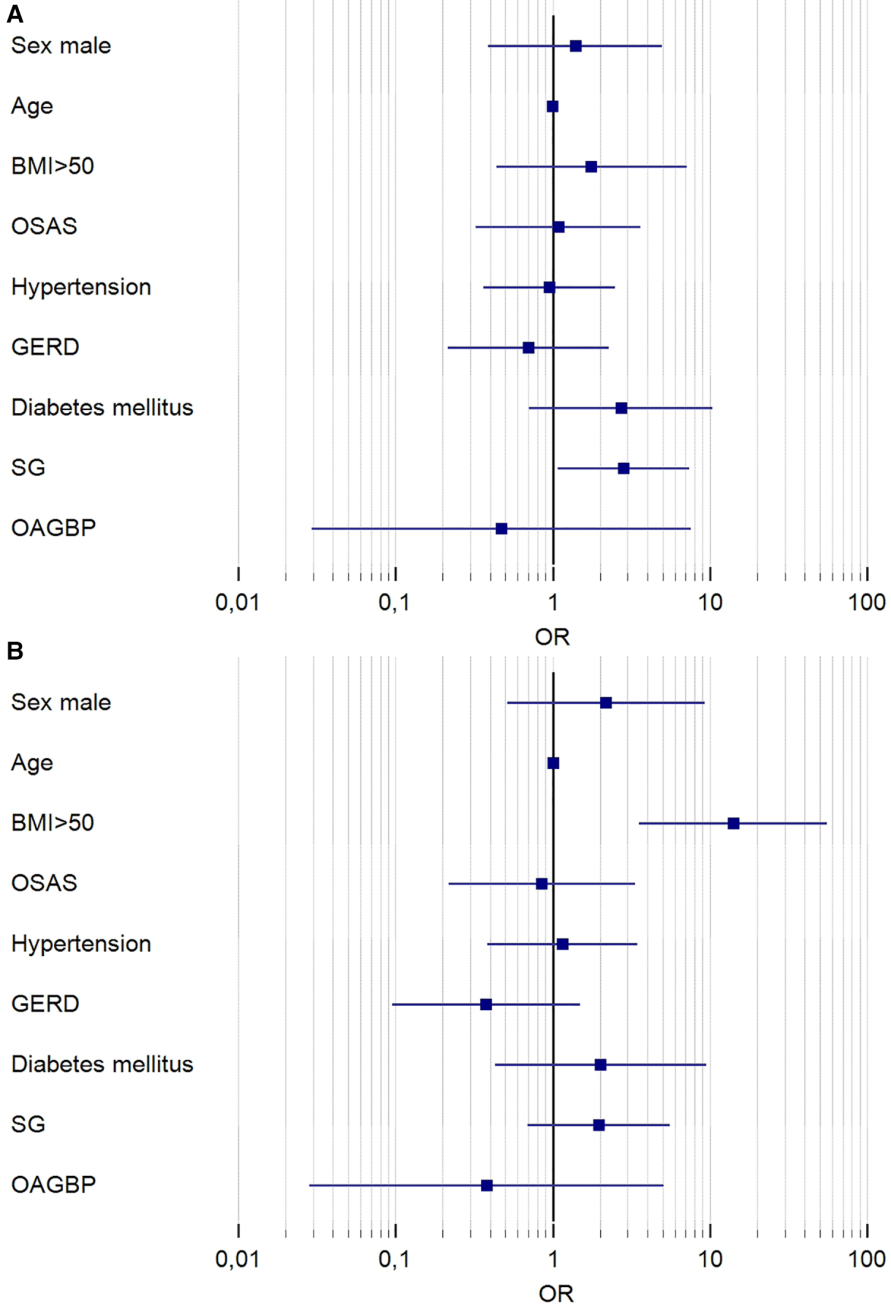
Forest plot of results of multivariate analysis. Results are reported in logarithmic scale. In (**A**) are reported the results of logistic regression considering the outcome of EWL > 50% at 24 months; the only significant variable predictive of surgical failure was SG (OR = 2.816; 95%CI = 1.0741–7.3837; *p* = 0.0353). In (**B**) are reported the results of logistic regression considering the outcome of BMI < 35 Kg/m^2^ at 24 months. The only significant variable predictive of surgical failure was superobesity (OR = 14.04; 95%CI = 3.5443–55.6861 *p* = 0.002). OR, odds ratio; OSAS, obstructive sleep apnea syndrome; GERD, gastroesophageal reflux disease; SG, sleeve gastrectomy; OAGBP, one-anastomosis gastric bypass.

At multivariate analysis (Figure 2), when considering surgical success as EWL > 50%, the only significant predictive factor for failure was SG (OR = 2.816; 95%CI = 1.0741–7.3837; *p* = 0.0353); indeed, when considering surgical success as BMI < 35, superobesity was an independent predictive factor for failure (OR = 14.04; *p* = 0.002).

Full comorbidity-related outcomes are reported in Table 4. Complete or partial resolution of OSAS was observed in 35 (67.3%) MO patients, and in 6 (54.5%) SO patients. Hypertension was completely or partially resolved in 36 (43.9%) MO patients, and in 3 (37.5%) SO patients. There were no significant differences among the groups.

**Table 4:** Partial or complete resolution of comorbidities at 24-month follow-up.

	MO patients	SO patients	*p*
OSAS	35/52 (67.3%)	6/11 (54.5%)	0.4939
Hypertension	36/82 (43.9%)	3/8 (37.5%)	1
GERD	42/70 (60%)	4/4 (100%)	0.2905
Diabetes mellitus	10/23 (43.5%)	1/2 (50%)	1
Dyslipidemia	12/60 (20%)	1/3 (33.3%)	0.5064

MO, morbidly obese; SO, superobese; OSAS, obstructive sleep apnea syndrome; GERD, gastroesophageal reflux disease.

Finally, we performed a comparison of the outcomes at 24 months of follow-up of patients that underwent SG (Table 5). The TWL and the EWL were significantly higher in the SO group, whereas no significant differences were observed regarding the EWL > 50%. Conversely, the BMI targets <35 Kg/m^2^ and <30 Kg/m^2^ were reached significantly more frequently by the MO group. The median BMI at 24 months was 30.3 (27.1–32.2) Kg/m^2^ in the MO group, and 38.4 (36.5–41.1) Kg/m^2^ in the SO group (*p* < 0.0001).

**Table 5:** Comparison of outcomes at 24 months of patients that underwent sleeve gastrectomy.

	Morbid obese patients (*n* = 62)	Superobese patients (*n* = 11)	*p*
TWL, Kg	26.1 ± 13.3	41.5 ± 20.6	0.0185
EWL, Kg (median, IQR)	37.4 (32–47.1)	87.2 (71.5–93.6)	<0.0001
EWL > 50%	45 (72.6%)	7 (63.6%)	0.7187
BMI < 35 Kg/m^2^	53 (85.5%)	1 (9.1%)	<0.0001
BMI < 30 Kg/m^2^	28 (45.2%)	0	0.0049
BMI, Kg/m^2^ (median, IQR)	30.3 (27.1–32.2)	38.4 (36.5–41.1)	<0.0001

TWL, total weight loss; EWL, Excess weight loss; BMI, body mass index.

## Discussion

In one of the most famous paradoxes of Zeno of Elea, Achilles, famous for his speed, will never reach the tortoise after granting her a head start. This narration appears to be similar to what happens to SO patients: in fact, SO patients start with a handicap (a greater BMI), run faster than MO patients (gaining a greater weight loss), but lose the race against them, rarely reaching the finish line of the BMI target <30 kg/m^2^.

In fact, our study confirmed that SO patients may present suboptimal results in terms of weight loss after bariatric surgery compared to MO patients. Our comparative analysis showed that SO patients significantly gained more weight loss during the first two years after surgery, but this was not enough to achieve surgical success in terms of BMI < 35 kg/m^2^: in fact, only 8 (42.1%) out of 19 SO patients reached this result, compared to 137 out of 154 (89%) MO patients. When considering the target of BMI < 30 kg/m^2^, only one SO patient (5.3%) reached the result, compared to 90 (58.4%) in the MO group. Furthermore, in our study, superobesity was identified as an independent risk factor for failure when considering the outcome of BMI < 35 kg/m^2^ at 24 months. However, we should note that, considering the outcome of EWL > 50% at 24 months, the results were not significantly different between the two groups. This result raises the issue whether EWL > 50% is a proper target to evaluate bariatric surgery failure, or if it would be better to consider the final BMI.

According to our results, several studies in which patients were stratified by obesity category showed that a higher baseline BMI was associated with a lower %EWL (2, 16, 17).

Our study did not show an increased risk of postoperative complications in SO patients. In this regard, the literature is still contrasting and inconclusive. Verhoeff reported five years of MBSAQIP data for SO patients (3). In his study, which included 751,952 obese patients who underwent obesity surgery, 173,110 (23%) SO patients had a small but significant increase in postoperative complications in nearly all measured domains, including serious complications (3.7% SO vs. 3.2% non-SO, *p* < 0.001) and mortality (0.17% SO vs. 0.07% non-SO, *p* < 0.001).

Tien-Chou Soong reported data of SO patients who underwent LRYGBP, OAGB, and SG: LRYGBP was associated with a higher major 30-day complication rate (4.8%) than other procedures (0.8% OAGB, *p* = 0.041; and 0.5% SG, *p* = 0.023) (4). Conversely, a recent meta-analysis compared SG and LRYGBP in SO patients, showing no difference in 30-day complications (2). Bettencourt-Silva did not report a difference in the 30-day morbidity rate comparing SG, LRYGBP and adjustable gastric banding (10). Only one study reported severe major adverse events in LRYGBP, even if this study showed no difference in 30-day complications (17).

Our univariate analysis on patients that underwent SG demonstrated that this type of intervention was associated to a lower success-rate in SO patients than in MO patients; furthermore, at multivariate analysis, we demonstrated that SG was an independent risk factor for surgical failure. SG is the most common procedure performed worldwide 18 in SO and non-SO patients. Verhoeff reported in the last five years that SG was used in 70% of SO patients and in 74.7% of non-SO patients (*p* < 0.001), and LRYGBP was used in 30% of SO patients and in 25.3% of non-SO patients (*p* < 0.001) (3).

A recent meta-analysis compared SG and LRYGBP in SO patients and showed that considerable weight loss was achieved following both procedures, but LRYGBP accomplished a higher %EWL (mean 59.73%) at 12 months (2). Although this tendency was already present at 6 months, a significant difference was not shown after 24 months; perhaps this result could be explained by the loss of patients at follow-up. Bettencourt-Silva included 213 SO patients in his study, performing 127 RYGB, 67 SG and 19 adjustable gastric banding procedures (10). At 12 and 24 months, the median %EWL was higher in LRYGBP (67.5% and 72.19%, respectively; *p* < 0.001) than in SG (58.7% and 59.9%) and adjustable gastric banding (38.7% and 48.3%). At 12 and 24 months, the median BMI was lower in LRYGBP (34.5 and 33.2 kg/m^2^) than in SG and adjustable gastric banding (*p* < 0.001). Tien-Chou Soong reported data on 498 SO patients, who underwent 62 RYGB, 190 SG, and 246 OAGB procedures (4). Five years after surgery, 64.6% of all the patients achieved a BMI < 35 kg/m^2^: 56.1% in the SG, 58.6% in the LRYGBP, and 71.8% in the OAGB group. Interestingly, the authors described LRYGBP with biliopancreatic limb of 100 cm and alimentary limb of 250–300 cm without common channel measurement, and OAGB with biliopancreatic limb of 250–350 cm without common channel measurement.

The choice of surgical procedure in SO patients should always be carefully evaluated and should be addressed based on surgical perioperative risk and surgical results (weight-loss, resolution of obesity-related complications, and quality of life). Considering weight-regain as the most common bariatric surgery failure in SO patients, reoperation could achieve better improvement in primary SG than primary RYGB, despite new procedures were promising (18).

Furthermore, it would be necessary to reconsider malabsorptive procedures as long limb LRYGBP, distal OAGB, single anastomosis gastro-ileal bypass (SAGI), single anastomosis duodeno-ileal bypass (SADI-S).

Furthermore, it would be necessary to reconsider mixed (restrictive and malbsorbitive) procedures as long limb LRYGBP, distal OAGB, single anastomosis gastro-ileal bypass (SAGI), single anastomosis duodeno-ileal bypass (SADI-S).

Our study has some limitations. First, this was a retrospective study. Then, the number of patients in the SO group was limited, and it is likely that the number was not sufficient to reach the statistical power to detect significant differences between the two groups regarding some aspects, including postoperative complications and comorbidity resolution. Furthermore, in our multivariate analysis SG was an independent risk factor for surgical failure, but the analysis was conducted considering both the SO and MO patients. For these reasons, the results of our study should be prudently considered.

## Conclusion

The management of SO patients is an important issue. Our study confirmed that, after surgery, patients with BMI > 50 kg/m^2^ tend to lose more weight than MO patients, but less frequently reach the goal of BMI < 35 kg/m^2^. Even if our study has several limitations, mainly the limited number of SO patients, SG seems to be associated with an increased risk of surgical failure, particularly in SO patients. In this setting, it could be necessary to re-consider mixed procedures; however, the type of intervention should be chosen based on the patient's characteristics, according to the rule that “one size does not fit all”.

## Data Availability

The raw data supporting the conclusions of this article will be made available by the authors, without undue reservation.
